# A comparison of the clinical effects of arthroscopic treatment for popliteal cyst between techniques using one posteromedial portal and two posteromedial portals

**DOI:** 10.1097/MD.0000000000020020

**Published:** 2020-05-15

**Authors:** Dongsheng Guo, Liang Cheng, Guangxiang Chen, Xiao Yu, Hong Zhang, Yuanshi She

**Affiliations:** a The Affiliated Suzhou Hospital of Nanjing Medical University; b Department of Orthopedic Surgery, The Affiliated Suzhou Hospital of Nanjing Medical University, Suzhou, Jiangsu, China.

**Keywords:** arthroscopy, magnetic resonance imaging, posteromedial portal, surgery

## Abstract

There is a lack of research comparing the clinical effects of arthroscopic treatment of popliteal cysts between the one posteromedial portal (OPP) technique and the two posteromedial portals (TPP) technique. The aim of this study was to evaluate and compare the clinical efficacy of arthroscopic treatment for popliteal cysts between the 2 techniques.

Patients with symptomatic popliteal cysts after surgery were retrospectively invited to participate in this study. They received arthroscopy treatment via the OPP technique or the TPP technique. At the final follow-up, the Rauschning and Lindgren criteria and the Lysholm score were used for clinical evaluation. Moreover, magnetic resonance imaging was performed to detect the recurrence of cysts postoperatively.

Finally, 53 patients with symptomatic popliteal cysts were included in this study, including 25 in the OPP group and 28 in the TPP group. The operation time of the TPP group was significantly longer than that of the OPP group (*P* < .001). In the OPP group, the cysts disappeared in 17 patients and reduced in size in 8 patients. In the TPP group, the cysts disappeared in 23 patients and reduced in size in 5 patients. According to the Rauschning and Lindgren classification, the recurrence rate was significantly lower in the TPP group (0%) than in the OPP group (4%) (*P* = .03). In addition, there was no significant difference in the Lysholm score between the OPP group and the TPP group (*P* = .77).

TPP technique is more effective and superior than OPP technique for the treatment of popliteal cysts.

## 1 Introduction

The popliteal cyst, also known as Backer cyst, is a frequent condition among patients between 35 and 70 years old, and most of the cases are associated with chronic arthropathies.^[1]^ Popliteal cyst is mostly located in the medial aspect of the popliteal fossa.^[2]^ Previous studies on the pathogenesis of popliteal cyst have described that they are associated with intraarticular pathology and valvular slit mechanisms.^[3,4,5]^ When the popliteal bursa is present, it becomes symptomatic by responding to the intraarticular disease because of its continuity with the knee joint.^[6]^

Open excision of popliteal cysts is traditionally performed in symptomatic cases. However, the open procedure entails a large incision on the posterior side of the knee, a risk of neural injuries, a higher recurrence rate, longer time to perform, and more perioperative complications.^[7,8,9,10]^ Previously, popliteal cystectomy is mostly done under open surgery. However, almost half of the cases would have recurrence after open procedure.^[11]^ The main reason was due to the unsolved intraarticular pathological conditions (such as meniscus injuries, patellofemoral arthritis, rheumatoid arthritis, synovitis, cartilage damage, etc).^[5,12,13]^ With the development of arthroscopic technique, popliteal cystectomy via arthroscopy becomes much promising.^[14]^ Compared with open excision, arthroscopic treatment for popliteal cysts is minimally invasive, associated with a lower risk of poor outcome, directly addresses both intraarticular pathology and the popliteal cyst, and exhibits better clinical outcomes.^[15,16,17]^

Based on the 1-way valvular slit mechanism, the key to successful treatment of popliteal cysts is dealing with the associated intraarticular lesions and arthroscopic interventions.^[18,19]^ Sansone et al^[20]^ debrided popliteal cysts using one posteromedial portal (OPP), and they observed optimal or good clinical results in 95% of the patients treated at 2 years after surgery. Additionally, Ahn et al^[21]^ reported popliteal cystectomy effectively by using an posteromedial portal. Jiang et al^[22]^ also reported the posteromedial portal method yielded an excellent result without popliteal cyst recurrence. Gu et al^[23]^ used two posteromedial portals (TPP) to perform complete popliteal cystectomy. Thus far, there is a lack of research comparing the clinical effects of arthroscopic treatment of popliteal cysts between the OPP technique and the TPP technique.

Therefore, the purpose of this study was to retrospectively investigate the clinical results of arthroscopic treatment for popliteal cysts via OPP or TPP and to investigate whether it is necessary to create 2 portals to treat popliteal cysts. It was hypothesized that arthroscopic treatment for popliteal cysts via TPP would improve knee functional recovery for the treatment of popliteal cysts.

## 2 Methods

### 2.1 Participants

This retrospective study was approved by the Health Sciences Institutional Review Board of our hospital, and written consent was obtained from all participants. From January 2013 to January 2017, patients scheduled for popliteal cyst resection under arthroscopy were invited to participate in this study. All of these patients had mass-like symptoms, such as swelling, pain, and limited motion of the knee joint. The inclusion criteria were as follows:

(1) patients undergoing unilateral popliteal cyst resection
(2) patients aged 18 to 50 years
(3) patients scheduled for surgery via an arthroscopic resection technique.

The exclusion criteria were as follows:

(1) patients scheduled for open surgeries
(2) patients undergoing combined ligament reconstruction
(3) patients undergoing revision surgery, and
(4) patients with osteoarthritis.

### 2.2 Surgical technique

#### 2.2.1 Arthroscopy via OPP

**Figure 1:**
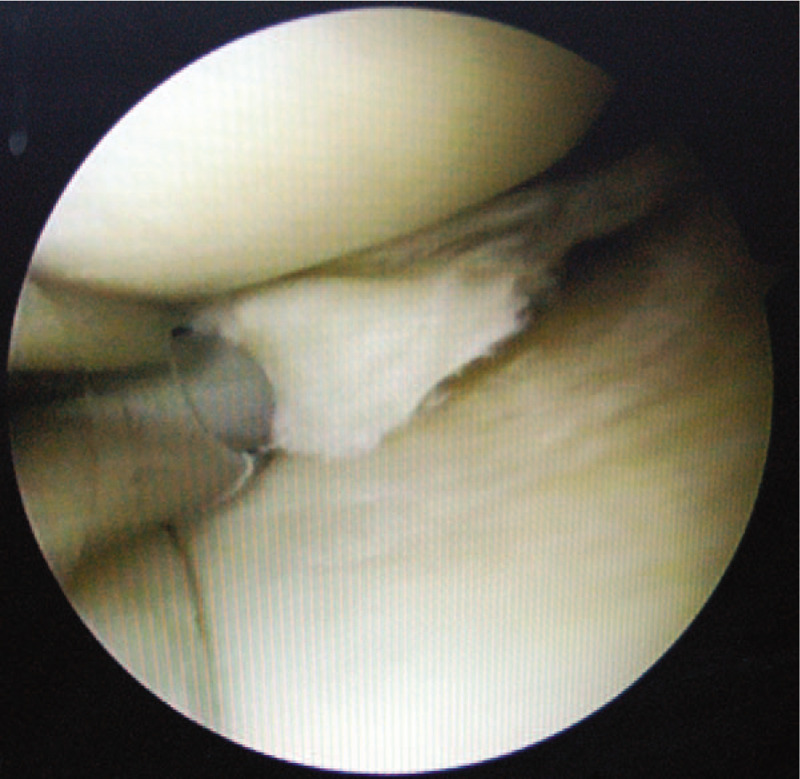
The intraarticular pathologie (meniscual tear) was treated under arthroscopy.

**Figure 2:**
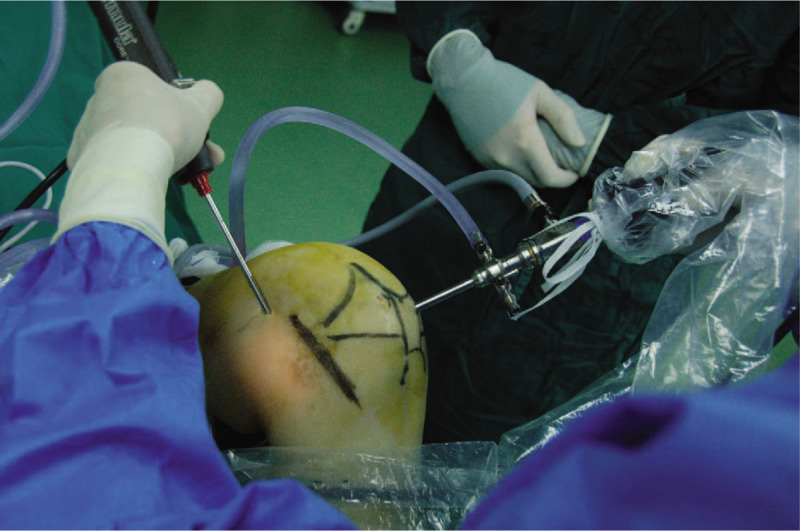
Surgical image showed arthroscopic technique via one posteromedial portal.

**Figure 3:**
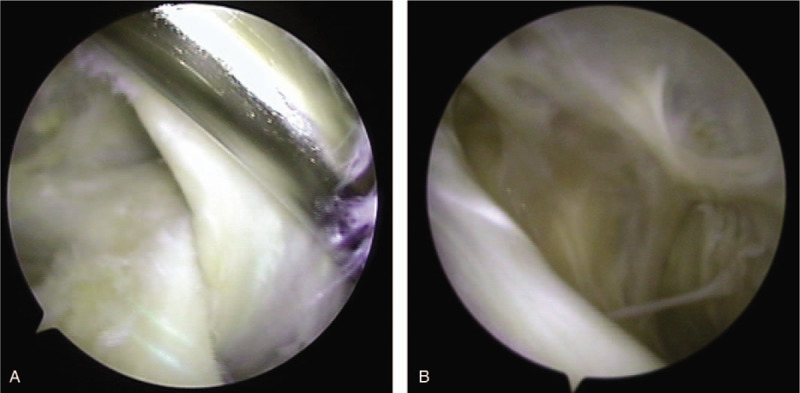
(A) Arthroscopic image of 1 portal technique. The opening of the cyst overlaid the posteromedial side of the medial head of gastrocnemius; (B) Arthroscopic image of 1 portal technique. Observation of the cyst wall.

Patients underwent arthroscopic surgery under general or spinal anesthesia in the supine position. Routine arthroscopic examination of the knee joint was performed through the anterolateral portal. After examining and treating the intraarticular pathologies (such as meniscus tear, synovitis, loose body, etc) (Fig. 1), the arthroscope was redirected toward the posteromedial compartment through the intercondylar notch. One spinal needle was inserted into the posteromedial compartment with the knee flexed at 90° to create the first posteromedial portal (Fig. 2). The capsular fold was resected. The posteromedial side of the medial head of gastrocnemius was identified and located. In general, the opening of the cyst overlaid the posteromedial side of the medial head of gastrocnemius and was sometimes obscured by a membrane.^[5,24]^ Then, the valvular structure was enlarged using a shaver from the posteromedial portal. The sign of a successful opening was that a large amount of bright yellow fluid flowed into the joint cavity (Fig. 3). Finally, after the end of all involved procedures, the incision was closed, and a compressive dressing was applied.

#### 2.2.2 Arthroscopy via TPP

**Figure 4:**
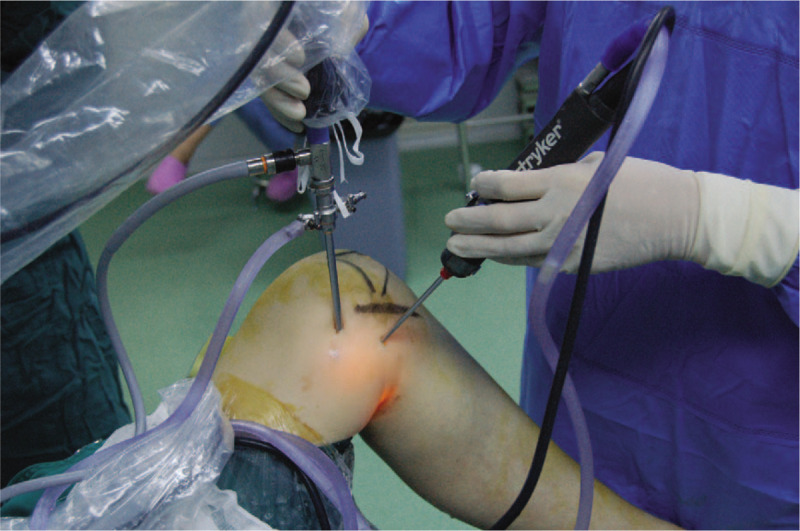
Surgical image showed arthroscopic technique via two posteromedial portals.

**Figure 5:**
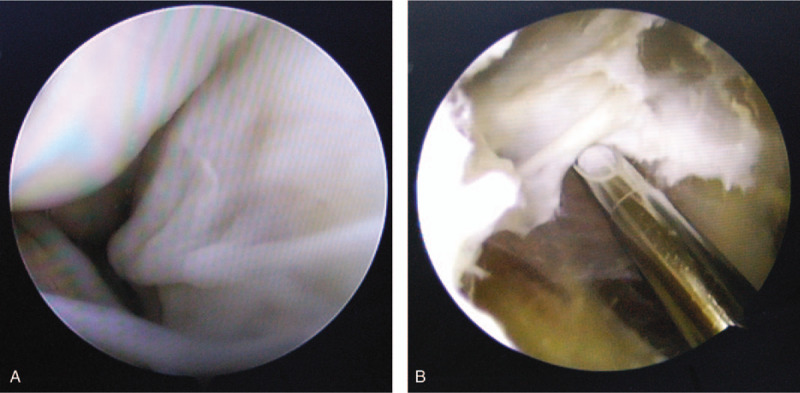
(A)arthroscopic image of 2 portals technique. Observation of the cyst wall; (B) Arthroscopic image of 2 portals technique. The inner wall of the cyst was resected using a low-suction shaver.

Routine arthroscopic detection and intraarticular pathology treatment were performed in the same way as described in the OPP group. The first posteromedial portal was established as described above. The capsular fold was resected via the posteromedial portal, and the medial head of the gastrocnemius was exposed. A switching stick was inserted into the cystic inner cavity from the first posteromedial portal. Then, the arthroscope was advanced to the cyst wall using the switching stick. A second posteromedial portal was created as an operating portal approximately 4 cm distal to the first portal along the longitudinal axis of the tibia (Fig. 4). The inner wall of the cyst was resected using a low-suction shaver. Once the adipose tissue was revealed, there were nerves and blood vessels nearby (Fig. 5). Finally, the incision was closed, and a compressive dressing was applied.

### 2.3 Rehabilitation

All of the patients were postoperatively rehabilitated according to the same protocol. From the day after the operation, lower extremity quadriceps contraction training, and ankle pump movements were started. Weight bearing was permitted depending on the intraarticular pathology or lesion treatment. For the patients with meniscoplasty, loose body removal or synovitis debridement, and weight bearing was allowed at the second day after surgery as tolerated. For the patients with meniscorepair, weight bearing was allowed at 2 to 4 weeks after surgery, and then weight bearing was allowed as tolerated with 2 crutches.

### 2.4 Clinical evaluation

**Figure 6:**
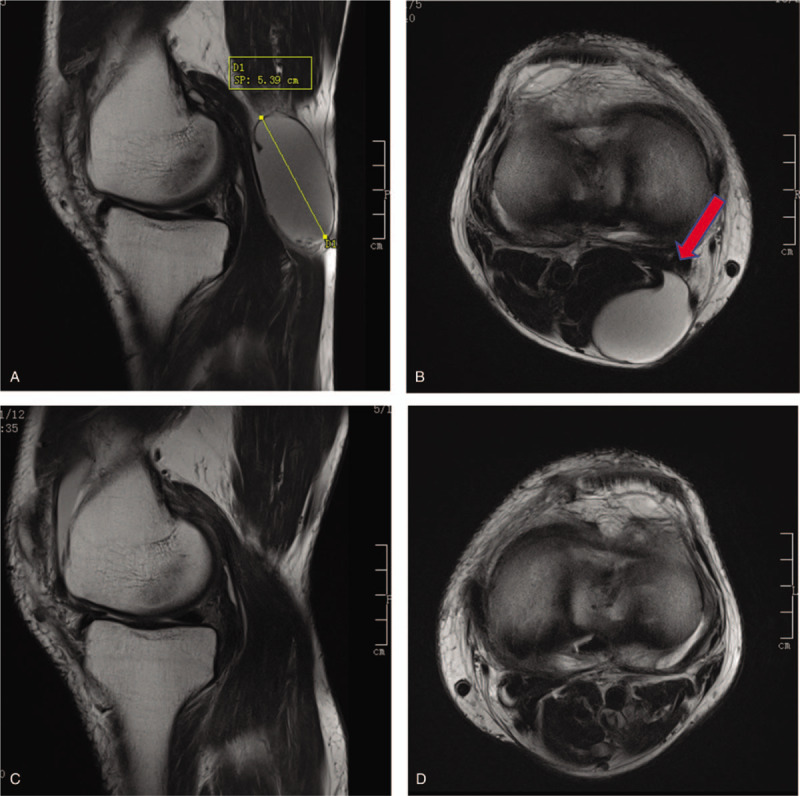
(A) large popliteal cyst detected in sagittal T2; (B) large popliteal cyst detected in axial T2 view; (C) The sagittal view revealed disappearance of the cyst after operation; (D) The axial view of the knee after operation.

The clinical and radiological data were collected from admission records, operative records, and the Picture Archiving and Communication System. Patient demographics, including age, sex, and combined lesions, were collected. Operating time was also collected. Magnetic resonance imaging (MRI) was performed preoperatively to detect the cyst location and its diameter with combined intraarticular pathology and postoperatively to confirm the complete removal of the cyst (Fig. 6). In addition, a follow-up MRI was performed to detect the recurrence of cysts at least 12 months postoperatively. The Rauschning and Lindgren grades and the Lysholm score were evaluated. According to the Rauschning and Lindgren classification, the patients with grade II and above were defined as recurrent patients.^[11]^ The complications were also documented.

### 2.5 Statistics analysis

The data are presented as the mean ± standard deviation. The statistical significance of differences in continuous data was compared using an independent t test or Mann–Whitney *U* test. The dichotomous variables were compared using the Pearson Chi-squared test or Fisher exact probability test. All statistical data are 2-sided and were evaluated at a 5% level of significance. All statistical analyses were performed with SPSS software (Version 17.0; SPSS, Chicago, IL).

## 3 Results

In total, 53 patients with popliteal cysts were investigated. Twenty-five patients received arthroscopy via OPP (the OPP group), and 28 patients received arthroscopy via TPP (the TPP group). There were no significant differences in age, gender, cyst diameter preoperative Lysholm score, or preoperative Rauschning and Lindgren grade between the 2 groups **(**Table 1**)**.

**Table 1:**
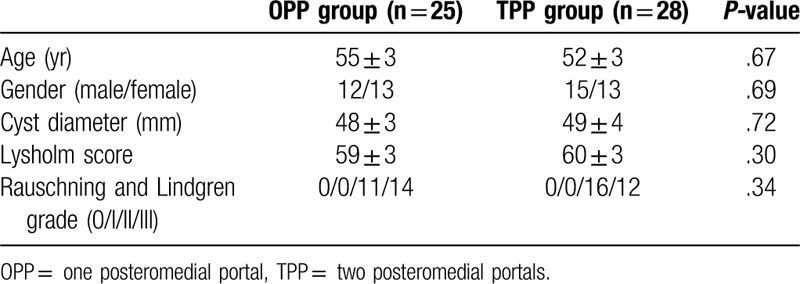
Demographic characteristics and perioperative data.

Among the cohort of patients, 36 (68%) had meniscal lesions, including 17 patients in the OPP group and 19 patients in the TPP group. Degenerative osteoarthritis was observed in 33 patients (62%) in total, including 15 patients in the OPP group and 18 patients in the TPP group. Synovitis was also recorded in 28 patients (53%), including 13 patients in the OPP group and 15 patients in the TPP group. Moreover, 1 patient in the TPP group had plica. There was no significant difference in the intraarticular lesion rate between the OPP group and the TPP group (*P* > .05).

**Figure 7:**
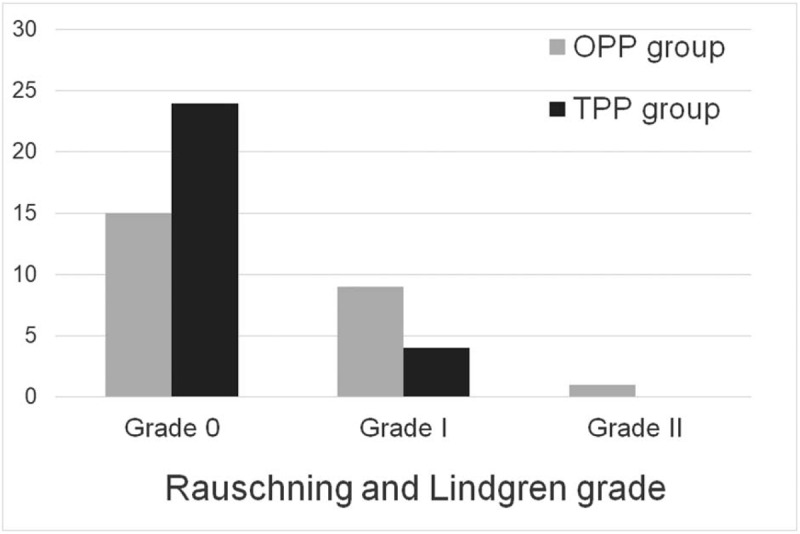
Comparison of Rauschning and Lindgren grade between one posteromedial portal technique and two posteromedial portals technique.

According to the operation record, the operation time of the TPP group was significantly longer than that of the OPP group (48.3 ± 5.5  vs 38.4 ± 6.9 minutes; *P* < .001). Fifty-three cases all received an MRI scan at their last follow-up. In the OPP group, the cysts disappeared in 17 cases and reduced in size in 8 cases. In the TPP group, the cysts disappeared in 23 cases and reduced in size in 5 cases. Regarding the Rauschning and Lindgren grade, 15 patients were graded as Grade 0, 9 patients were graded as Grade I, and 1 patient was graded as Grade II in the OPP group. In the TPP group, 24 patients were graded as Grade 0, and 4 patients were graded as Grade I (Fig. 7). According to the Rauschning and Lindgren classification, the recurrence rate was significantly lower in the TPP group (0%) than in the OPP group (4%) (*P* = .03). In addition, there was no significant difference in Lysholm score between the OPP group and the TPP group (86.5 ± 5.9 vs 86.1 ± 3.9; *P* = .77).

Regarding the complications, 1 patient had a deep vein thrombosis due to lack of functional exercise and recovered after conservative treatment. This patient was obese and did not perform regular quadriceps femoris functional exercises or ankle pump exercises after the operation. One patient had hypoesthesia on the medial shank, which was completely recovered after treatment by neurotrophic medicine.

## 4 Discussion

As reported, arthroscope can directly address the cyst as well as the posterior transverse synovial infold.^[25]^ Some surgeons perform direct arthroscopic excision using a 70° arthroscope and posteromedial portal for the treatment of popliteal cysts.^[12]^ Although the 70° arthroscopy with only OPP has a large viewing range, there are still visual blind spots. Therefore, the risk of recurrence still persists because arthroscopy may not allow complete surgical excision.^[26]^ In some cases, incomplete removal occurred because the cyst wall could not be clearly identified. In the present study, we performed both the 1 portal technique and the 2 portals technique to treat popliteal cysts with a 30° arthroscope. It was found that 2 portals technique was more efficiency as there was no recurrence. A 30° arthroscopy with TPP allows for an improved horizon and visualization over arthroscopic approaches and achieves complete cyst excision while minimizing damage to the soft tissues. In the TPP group, most cysts were resected completely, as confirmed by MRI.

In the present study, the cysts disappeared in 17 patients and reduced in size in 8 patients in the OPP group. In the TPP group, the cysts disappeared in 23 patients and reduced in size in 5 patients. According to the Rauschning and Lindgren classification, the recurrence rate was significantly lower in the TPP group (0%) than in the OPP group (4%). Previously, Xinxian et al^[10]^ reported that popliteal cysts disappeared in 17 (55%) patients, were reduced in 9 (29%) patients and persisted in 5 (16%) patients in the arthroscopic decompression group (1 portal technique). Yang et al^[11]^ reported that the recurrence rate of arthroscopic decompression is 3.1%. Furthermore, Ahn et al^[27]^ resected the capsular fold through the posteromedial working portal and used the posteromedial cystic portal for complete cyst removal. The follow-up MRI study showed that the cyst had disappeared in 17 knees (55%) and had reduced in size in 14 knees (45%) among the 31 patients. Jiang et al^[18]^ performed popliteal cyst debridement through 2 portals, and they reported that all patients had no recurrence at the follow-up after surgery. Ohishi et al^[28]^ performed arthroscopic cyst decompression via 2 posterior portals (posterolateral portal and posteromedial portal) after creating a transseptal portal and reported that 12 cysts disappeared completely, while 16 reduced and 1 enlarged in size by the time of the final follow-up.

Previously, Han et al^[29]^ reported complications after arthroscopic treatment for popliteal cysts included hematoma formation, portal site infection and discomfort, persistent pain and swelling, and technical error during cyst debridement. Previously, Kp et al^[30]^ reported 1 case of popliteal artery aneurysm after arthroscopic cystectomy of a popliteal cyst, which is an uncommon complication. They recommended not shaving the lateral aspect of the cyst while performing arthroscopic cystectomy when MRI revealed that the popliteal artery was close to the cyst. In the present study, 2 patients (3.8%) had complications, including one who developed deep vein thrombosis and one with hypoesthesia. One patient developed deep vein thrombosis due to high body mass index. In cases such as this, early quadriceps femoris exercises and ankle pump exercises are encouraged after operation. Anticoagulants should also be used 6 hours after the operation to prevent deep venous thrombosis.

Our study has some limitations. The follow-up time was a little short, only 1 to 2 years. We do not know whether these cysts will recur over a longer term. In addition, there were only 53 cases in our study. Next, we will collect more cases to increase the statistical power. Further studies with long-term follow-up and more cases are warranted to guide the treatment recommendation.

## 5 Conclusions

Arthroscopic excision of the popliteal cyst using 30° arthroscopy with TPP can correct the valvular mechanism of the capsular fold. Arthroscopic management via TPP is an effective and superior method for the treatment of popliteal cysts, with a small incision, quick recovery, good efficacy, and a low recurrence rate.

## Author contributions

DSG, LC, GXC, XY, and HZ performed the investigations. YSS gave the guidance during this study. DSG, LC, and YSS analyzed and interpreted the data regarding the MRI results, and were a major contributor in writing the manuscript. All authors read and approved the final manuscript

## References

[1] Maximiliano VJ Matias PD Pablo ZJ Infected Bakerʼs cyst: a new classification, diagnosis and treatment recommendations. J Orthop Case Rep 2018;8:16–23. 10.13107/jocr.2250-0685.1238 PMC6424320 30915286

[2] Zhou XN Li B Wang JS Surgical treatment of popliteal cyst: a systematic review and meta-analysis. J Orthop Surg Res 2016;11:22. 26879283 10.1186/s13018-016-0356-3 PMC4754995

[3] Calvisi V Zoccali C Arthroscopic patterns of the poster-medial aspect of the knee joint: classification of the gastrocnemius-semimembranosus gateway and its relationship with Baker's cyst. Muscles Ligaments Tendons J 2016;6:492–8. 28217572 10.11138/mltj/2016.6.4.492 PMC5310751

[4] Rupp S Seil R Jochum P Popliteal cysts in adults. Prevalence, associated intraarticular lesions, and results after arthroscopic treatment. Am J Sports Med 2002;30:112–5. 11799006 10.1177/03635465020300010401

[5] Kim KI Lee SH Ahn JH Arthroscopic anatomic study of posteromedial joint capsule in knee joint associated with popliteal cyst. Arch Orthop Trauma Surg 2014;134:979–84. 24781525 10.1007/s00402-014-2001-0

[6] Johnson LL van Dyk GE Johnson CA The popliteal bursa (Baker's cyst): an arthroscopic perspective and the epidemiology. Arthroscopy 1997;13:66–72. 9043606 10.1016/s0749-8063(97)90211-5

[7] Takahashi M Nagano A Arthroscopic treatment of popliteal cyst and visualization of its cavity through the posterior portal of the knee. Arthroscopy 2005;21:638. 15891743 10.1016/j.arthro.2005.02.007

[8] Snir N Hamula M Wolfson T Popliteal cyst excision using open posterior approach after arthroscopic partial medial meniscectomy. Arthrosc Tech 2013;2:e295–8. 24266002 10.1016/j.eats.2013.04.001 PMC3834643

[9] Saylik M Gokkus K Treatment of baker cyst, by using open posterior cystectomy and supine arthroscopy on recalcitrant cases (103 knees). BMC Musculoskelet Disord 2016;17:435. 27756267 10.1186/s12891-016-1291-5 PMC5069796

[10] Xinxian X Yuezheng H Jian L Clinical outcome of arthroscopic management of popliteal cysts with or without additional posterior open cystectomy. Orthopade 2018;47:530–5. 29691604 10.1007/s00132-018-3573-0

[11] Ravlic-Gulan J Gulan G Novak S Rapid recurrence of a giant popliteal cyst in a patient with rheumatoid arthritis. J Clin Rheumatol 2009;15:300–2. 19734737 10.1097/RHU.0b013e3181b5c653

[12] Herman AM Marzo JM Popliteal cysts: a current review. Orthopedics 2014;37:e678–84. 10.3928/01477447-20140728-52 25102502

[13] Chatzopoulos D Moralidis E Markou P Baker's cysts in knees with chronic osteoarthritic pain: a clinical, ultrasonographic, radiographic and scintigraphic evaluation. Rheumatol Int 2008;29:141–6. 18584176 10.1007/s00296-008-0639-z

[14] Calvisi V Lupparelli S Giuliani P Arthroscopic all-inside suture of symptomatic Baker's cysts: a technical option for surgical treatment in adults. Knee Surg Sports Traumatol Arthrosc 2007;15:1452–60. 17671780 10.1007/s00167-007-0383-z

[15] Yang B Wang F Lou Y A comparison of clinical efficacy between different surgical approaches for popliteal cyst. J Orthop Surg Res 2017;12:158. 29070055 10.1186/s13018-017-0659-z PMC5657075

[16] Cho JH Clinical results of direct arthroscopic excision of popliteal cyst using a posteromedial portal. Knee Surg Relat Res 2012;24:235–40. 23269962 10.5792/ksrr.2012.24.4.235 PMC3526761

[17] Kongmalai P Chernchujit B Arthroscopic treatment of popliteal cyst: a direct posterior portal by inside-out technique for intracystic debridement. Arthrosc Tech 2015;4:e143–8. 26052491 10.1016/j.eats.2014.12.002 PMC4454833

[18] Sansone V Sosio C da Gama Malcher M An unusual cause of popliteal cyst. Arthroscopy 2004;20:432–4. 15067286 10.1016/j.arthro.2004.01.015

[19] Yang JH Kwon HH Lee JK Successful arthroscopic treatment of refractory and complicated popliteal cyst associated with rheumatoid arthritis in combination with osteoarthritis: case series and literature review. Rheumatol Int 2019;39:2177–83. 30976834 10.1007/s00296-019-04278-9

[20] Sansone V De Ponti A Arthroscopic treatment of popliteal cyst and associated intraarticular knee disorders in adults. Arthroscopy 1999;15:368–72. 10355711 10.1016/s0749-8063(99)70053-8

[21] Ahn JH Yoo JC Lee SH Arthroscopic cystectomy for popliteal cysts through the posteromedial cystic portal. Arthroscopy 2007;23:559e1–4. 17478291 10.1016/j.arthro.2006.07.050

[22] Jiang J Ni L Arthroscopic internal drainage and cystectomy of popliteal cyst in knee osteoarthritis. J Orthop Surg Res 2017;12:182. 29169352 10.1186/s13018-017-0670-4 PMC5701350

[23] Gu H Bi Q Chen J Arthroscopic treatment of popliteal cyst using a figure-of-four position and double posteromedial portals. Int Orthop 2019;43:1503–8. 30088053 10.1007/s00264-018-4087-4

[24] Frush TJ Noyes FR Baker's cyst: diagnostic and surgical considerations. Sports Health 2015;7:359–65. 26137182 10.1177/1941738113520130 PMC4481672

[25] Brazier BG Sudekum SA DeVito PM Arthroscopic treatment of popliteal cysts. Arthrosc Tech 2018;7:e1109–14. 30533356 10.1016/j.eats.2018.07.006 PMC6262067

[26] Ko S Ahn J Popliteal cystoscopic excisional debridement and removal of capsular fold of valvular mechanism of large recurrent popliteal cyst. Arthroscopy 2004;20:37–44. 14716277 10.1016/j.arthro.2003.10.017

[27] Ahn JH Lee SH Yoo JC Arthroscopic treatment of popliteal cysts: clinical and magnetic resonance imaging results. Arthroscopy 2010;26:1340–7. 20869836 10.1016/j.arthro.2010.02.012

[28] Ohishi T Takahashi M Suzuki D Treatment of popliteal cysts via arthroscopic enlargement of unidirectional valvular slits. Mod Rheumatol 2015;25:772–8. 25661740 10.3109/14397595.2015.1008779

[29] Han JH Bae JH Nha KW Arthroscopic treatment of popliteal cysts with and without cystectomy: a systematic review and meta-analysis. Knee Surg Relat Res 2019;31:103–12. 30893988 10.5792/ksrr.18.068 PMC6561675

[30] Kp V Yoon JR Nha KW Popliteal artery pseudoaneurysm after arthroscopic cystectomy of a popliteal cyst. Arthroscopy 2009;25:1054–7. 19732646 10.1016/j.arthro.2009.05.005

